# The innate immune response in SARS-CoV2 infection: focus on toll-like receptor 4 in severe disease outcomes

**DOI:** 10.3389/fimmu.2025.1658396

**Published:** 2025-10-07

**Authors:** Enrico Maggi, Nadine Landolina, Francesca Romana Mariotti, Enrico Munari, Nicola Tumino, Paola Vacca, Bruno Azzarone, Lorenzo Moretta

**Affiliations:** ^1^ Tumor Immunology Unit, Bambino Gesù Children’s Hospital, IRCCS, Rome, Italy; ^2^ Department of Pathology and Diagnostics, University and Hospital Trust Verona, Verona, Italy; ^3^ Innate Lymphoid Cells Unit, Bambino Gesù Children’s Hospital, IRCCS, Rome, Italy

**Keywords:** innate immunity, NK cells, SARS-CoV2 infection, spike glycoprotein, TLR4

## Abstract

Innate immunity is the first line of defense against infections, including the detection and response to SARS-CoV-2. Cells of the innate system are usually activated within hours after pathogen exposure and do not generate conventional immunological memory. In this review, the current knowledge of the innate immune cells and of pattern-recognition receptors in sensing and responding to SARS-CoV-2 to mount a protective response has been shortly reviewed. Subsequently, the evasion strategies of the virus, as the inhibition of IFN-I/III production and autophagic response, counteracting the innate cell activity (including NK cells), have been briefly outlined. In the course of the infection, these strategies are also capable of rendering dysfunctional most innate cells, thus deeply interfering with the onset and maintenance of adaptive immunity. Possible mechanism(s) for the maintenance of dysfunctional innate immune response are also discussed. In this context, the importance of a rapid and robust activation of innate immunity through toll-like receptor (TLR) 4 as a key paradigm central to host defense against COVID-19 pathogenesis is also illustrated. We also discuss how the viral excess plus inflammatory signals upregulating TLR4 on innate cells may initiate a vicious loop which maintains and improves hyperinflammation, leading to the most critical outcomes. Targeting the TLR4 or its signaling pathway may be a promising therapeutic strategy, offering the dual benefits of viral suppression and decreasing inflammation.

## 1 Introduction

COVID-19 pandemic has been caused by the β-coronavirus SARS-CoV-2, that had a dreadful impact, resulting in more than seven million documented deaths worldwide and four hundred million (underestimated) of Long-COVID cases as far as 2024 (1). Although originally defined as a respiratory viral infection, COVID-19 is now clearly recognized as a more complex, multistep, multi-organ immune-mediated disease. The virus infects primarily the cells of the upper- and then of the lower respiratory tract, triggering a wide spectrum of clinical manifestations, from asymptomatic, mild, and moderate to severe and critical symptoms (2). While most SARS-CoV-2 infections are mild, some patients develop uncontrolled inflammatory cell death, systemic inflammation with severe cytokine storm (a general term applied to maladaptive cytokine release in response to infection and other stimuli) (3), pneumonia, acute respiratory distress syndrome -ARDS-, thrombosis, and multiorgan failure with fatal outcomes (4–6).

The innate immune system plays a primary role against infections, including SARS-CoV-2. It is usually activated within hours after pathogen exposure and does not generate immunological memory. It can be distinguished in immediate or induced innate immunity. The former is rapidly activated (0–4 h) and relies on the activity of preformed soluble antimicrobial molecules, including antimicrobial enzymes and complement (C‘) system proteins. The induced innate immunity begins later (4–72 h), involves the activation and the recruitment of cells (as neutrophils, monocytes, macrophages and natural killer -NK cells), and lasts few days after the first exposure to pathogens. In this phase, innate cells can mount a process of resistance to reinfection, termed “trained immunity”, which involves structural chromatin modifications, alterations in DNA methylation, histone acetylation, upregulation of inflammation-related genes and changes in metabolic intermediates. This “trained immunity” provides the innate system with a memory-like activity, allowing to respond more effectively to re-exposure to pathogens (7).

In this review, we will briefly examine the current knowledge of innate immune cells and pattern-recognition receptors (PRRs) in sensing and responding to SARS-CoV-2 as well as evasion strategies of the virus counteracting the innate immunity. We will focus on mechanism(s) for maintenance of dysfunctional innate immune cells through the upregulation/activation of toll-like receptor (TLR) 4 and leading to the most severe outcomes.

## 2 SARS-CoV-2 structure and activation of the innate immune response

### 2.1 SARS-CoV-2 genome and virus cell cycle

SARS-CoV-2 is made up of an enveloped structure containing a genome of approximately 30 kb, constituted by single-stranded RNA (ss-RNA) encoding 29 proteins with diverse functions (8). Four are structural virion components such as the spike (S), envelope (E), membrane (M), and nucleocapsid (N) proteins (9). The S glycoprotein (SP), assembled into homotrimers on virion particles, mediates viral entry by attaching to and fusing with the host cell membrane (10). SP is cleaved by convertases (as transmembrane serine protease type II -TMPRSS2-, cathepsins, furin or metalloproteases), into a mature protein formed by the two non-covalently associated S1 and S2 subunits (11). S1 consists of the amino-terminal (NTD), the receptor-binding (RBD), and two carboxy-terminal (CTDs) domains protecting the inner S2 subunit.

S1 binds the receptor Angiotensin-Converting Enzyme 2 (ACE2) through the RBD, while S2 links the cell membrane allowing viral entry (12). Endocytosis is another viral entry modality involving ACE2 plus other co-factors (as HSPG, PS receptors, NRP, CD147, C-type lectins) whose mechanism is partially defined. ACE2 is scarcely expressed on circulating immune cells, while it is highly present on cells (as Monocytes, Dendritic cells -DC-, Epithelial cells, type 2 Pneumocytes, Alveolar macrophages, etc.) of tissues and organs, especially in the respiratory and digestive mucosa and myocardium (13), making these tissues more susceptible to infection.

Within the host cell, the viral genome encodes nine accessory proteins that promote viral shape. The genome is immediately translated by producing two long polyproteins (pp1a and pp1ab) which are cleaved by virus-encoded proteases to 16 nonstructural proteins (Nsp) that are devoted to assembly the replication–transcription complex, to modulate host cell compartments for generation of new virions and to release them through exocytosis. They are effective upon interaction with multiple genetically encoded PRRs (14).

### 2.2 Virus-mediated activation of immunity

#### 2.2.1 PRRs engagement

Thanks to the expression of different PRRs most of innate cells sense pathogen-associated- and damage-associated molecular patterns (PAMPs DAMPs) along the infection. Activated receptors drive the expression of pro-inflammatory cytokines, chemokines, adhesion molecules and interferons (IFNs), which recruit and activate other innate immune cells. This further amplifies the immune response and cell death to eliminate infected cells, promotes pathogen clearance, and, if the infection is not eradicated, activates adaptive immune response (15, 16). Among different PRRs, SARS-CoV-2 engages and triggers retinoic acid-inducible gene I (RIG-I)-like receptors (RLRs), TLRs, cGAS and stimulator of interferon genes (STING) pathway, the inflammasome nucleotide-binding oligomerization domain (NOD)-like- (NLRs)/absent in melanoma 2 like- AIM2 (ALRs) and C-type lectin (CLRs) receptors (17). SARS-CoV-2 proteins induce also mitochondria damage which release of mitDNA activating cGAS-STING pathway, contributing to IFN-β expression (18) and, in endothelial cells, to vascular damage and coagulopathy in patients with severe COVID-19 (19). Importantly, TLR, RLR, cGAS engagement have a prevalent anti-viral impact (beneficial in early clearing the virus but detrimental if stimulation persists), while NLRs/ALRs or CLRs triggering are essentially devoted to promote inflammation and apoptosis of infected cells (14, 20). Some extensive reviews on the PRRs and their signaling pathways activated by SARS-CoV-2 has been recently reported (14, 21–25).

#### 2.2.2 Autophagy induction

Macro-autophagy is part of the antiviral innate response (26, 27) and can be activated upon viral infection, stress sensing kinases, or triggered PRRs (28). During autophagy, cytoplasmic cargo, including viruses, is engulfed by double-layered membrane vesicles, named autophagosomes, and degraded upon fusion with lysosomes in a tightly regulated process that involves more than 30 autophagy-related proteins (ATGs) (29, 30). Following DNA/RNA virus entry, autophagy is the most active promoter of innate immunity through DAMP release. The molecular interplay between SARS-CoV-2 proteins and the immune-related process of autophagy is described in the section 2. Viral peptides derived from autophagic degradation are subsequently presented on MHC class I/II antigens by lymph nodal DCs to CD8+ and CD4+ T cells to initiate the virus-specific adaptive response (31).

#### 2.2.3 SARS-CoV-2 full protection

It involves a plethora of immune cells of both innate and adaptive immunity (32–34). Monocytes play the major role in the very early response being recruited into lymph nodes where they differentiate into immature-DCs and subsequently in mature DCs which present viral peptides to T cells (35). Primed CD8+ T cells home to the site of infection to directly kill infected cells or secrete antiviral cytokines as IFN-γ and tumor necrosis factor alpha (TNF-α). In the lymph nodal germinal center, CD4+ T follicular helper (Tfh) cells promote B cell affinity maturation and their activation into antibody-secreting cells (31). Monocytes, recruited into infected tissues as lung, differentiate into macrophages; when activated by the virus, they promote M1-like polarization and release of chemokines favoring homing/activation of circulating (NK/NKT cells and neutrophils) and other tissue effector cells as mucosal-associated invariant T -MAIT-, γδ T, innate lymphoid cells -ILCs-, T resident memory -Trm-. NK and virus-specific CD8+ T cells play the major role in limiting infection through their ability to lyse infected cells and produce antiviral cytokines. When infected cells and viral load are significantly reduced, monocytes/macrophages turn off inflammation by reducing effector cells through the production of anti-inflammatory/suppressive cytokines (IL-10, IL-27, IL-35, TGF.β) (32–34, 36).

### 2.3 SARS-CoV-2 variants of concern (VOCs)

VOCs are characterized by mutations in the viral genome, particularly in the SP playing a crucial role in the virus’s ability to infect host cells (**Table 1**). Variants such as Alpha (B.1.1.7), Beta (B.1.351), Gamma (P.1), Delta (B.1.617.2), and Omicron (B.1.1.529) have been identified and classified by the World Health Organization as VOCs due to their different transmissibility, virulence, pathogenicity, ability to induce Long-Covid-19 and resistance to neutralizing antibodies induced by vaccines (37). The Omicron variant demonstrated a substantial increase in transmissibility compared to previous strains, likely linked to the high number of SP mutations. Even though the Delta variant’s R0 was estimated to be very high (between 5 and 8), however, R0 of Omicron VOC was found to be even higher (approximately 3.19 times greater than that of Delta) (38, 39). Moreover, Omicron VOC displays lower pathogenicity, high immune escape potential and a significant reduction in Long COVID induction and vaccine efficacy for infection (37, 40). **Table 1** summarizes the main features of VOCs, including their interaction/activation of SP of each variant with TLR4/MD-2 complex (41).

**Table 1:** Structural and pathophysiological features of the main SARS-CoV-2 VOCs.

SARS-CoV-2 VOCs’ features		Wuhan strain	Alpha B.1.1.7	Beta B.1.351	Gamma P.1	Delta B.1.617.2	Omicron B.1.1.529	Ref
Spike protein mutations (Deletions)			8 (3)	9 (3)	11 (3)	7	30	(240)
S1 - TLR4 affinity (no. of hydrogen bonds - Modes of TLR4/MD2 dimerization)		+ (15 - 6)	++ (14 - 7)	+ (ND -ND)	+ (ND - 5)	+ (17 - 4)	+++ (16 - 3)	(41, 241)
Innate immunity: Pro- and anti-inflammatory cytokines	IL6, IL10, IL18, IL27	+++	+++	++	++	++	+/-	(242, 243)
	IFN-γ, IL-4	+/-	+	+	+	+	++	(243)
R0 range detected (vs Wuhan strain)		2.24-5.71	2,26-11,38 (> 35-45%)	(> 50%)	(> 150%)	3.2-8 (> 100%)	4.2 times > Delta VOC (> 250%)	(40, 240)
Immune escape			Moderate	High (due to E484K mutation)	Moderate (due to E484K mutation)	Moderate	High (due to multiple mutations in RBD as E484A, Q493K)	(37)
Pathogenicity (vs Wuhan strain)			Increased severity	No significant increase of severity	No significant increase of severity	Increased severity in unvaccinated people	Lower severity (risk only for not vaccinated people)	(37)
Association with Long-COVID	Olfactory dysfunctions	50% of infected people	50% of infected people	ND	ND	40% of infected people	17% of infected people	(244)
	Risk to develop Long Covid	++	++	ND	ND	++	+/-	(245, 246, 247)
Impact on vaccine efficacy (percent of protection vs Original mRNA vaccines)			90% Minor reduction in efficacy; Efficacy for severe disease	90% Reduction in neutralization by mRNAvaccines. Boosters recommend ed	90% Moderate reduction in efficacy. Boosters recommended	80% Reduced efficacy after one dose; full vaccination and boosters recommended	52% Reduction in vaccine efficacy for infection, boosters increase protection to severe disease	(37, 248, 249)

ND, not defined.

## 3 Evasion strategies of SARS-CoV-2 counteracting innate immunity

The principal function of the innate system is to induce an inflammatory response devoted to limiting viral replication. However, the virus may evolve some evasion strategies to suppress host defense: of note, more than 50% of the SARS-CoV-2-encoded proteins may counteract innate response (42, 43). SARS-CoV-2 has evolved distinct, sometimes overlapping, mechanisms to antagonize IFN production and autophagy at multiple levels, all promoting viral replication.

The major IFN antagonists are Nsp1, Nsp3, ORF6 and ORF3a/b/c. Nsp1 was shown to block the mRNA channel of the ribosome, turning off the expression of cytokines or IFN-stimulated antiviral genes in infected cells (44). By harnessing its de-ubiquitinase activity exerted by papain-like protease (PLP) 2 domain, Nsp3 inhibits various steps of PRR signaling (45, 46), cleaving the ubiquitin-like ISG15 protein, thus antagonizing MDA5 and IRF3 activation (47, 48). SARS-CoV-2 induces multiple proteins encoded in the Open Reading Frame (ORF) loci. ORF3a reduces STAT1 phosphorylation, the main transcription factor of IFN (49), while ORF3b and ORF3c inhibit type I IFN production by targeting mitochondrial antiviral-signaling protein (MAVS) for cleavage by caspase-3 (50). ORF6 disrupts nucleocytoplasmic trafficking by binding the nuclear pore Nup98-Rae1 complex, inhibiting STAT1 nuclear translocation (51, 52). Even though ORF6 alone is not sufficient to antagonize IFN pathway (53), its high levels could increase IFN resistance of SARS-CoV-2 VOCs compared to original strain (54–57).

SARS-CoV-2 infection leads to an incomplete/dysfunctional autophagy with a higher turnover of autophagosomes. Pharmacological activation of autophagy reduces replication of human Coronaviruses (huCoVs) and spreading (58).

ORF3a and ORF7a are the key viral components causing impaired autophagy (59–64), by preventing viral clearance and favoring viral replication by the accumulation of autophagosomes.

ORF3a interacts with autophagy process at different levels.: it prevents the fusion between autophagosomes and lysosomes, decreasing the autophagic flux and providing an immune escape from autophagy (42, 61, 63). In addition, it shows activity on endo-lysosomal compartments promoting lysosomal exocytosis and improving viral release (65). Lastly, ORF3a has been reported to counteract the flux, modulating the antiviral effect of the non-canonical STING1-mediated autophagy (66).

ORF7a dysregulates the late stages of autophagy by inhibiting the acidification of lysosomes (42, 67) and prevents autophagosome-lysosome fusion by promoting the degradation of the SNARE protein SNAP29 (62).

SARS-CoV-2 can also block autophagy turnover through the structural proteins M and E, which leads to the accumulation of autophagosomes and p62 in the cell (42, 67). Similarly, M and ORF10 are able to counteract innate immunity by promoting the autophagic degradation of MAVS (through mitophagy), which are important antiviral elements associated with mitochondria, leading to a reduction of type I IFN (IFNI) production (68, 69). Like ORF3a ORF10 also counteracts non-canonical autophagy by inhibiting STING1 activation (70).

Non-structural proteins also are able to downregulate autophagy: SARS-CoV-2 papain-like protease Nsp3 reduces the starvation-induced autophagy and disrupts the formation of the initiation complex that involves ULK1 and ATG13 (71). Nsp4 promotes accumulation of autophagosomes (42), while Nsp6 inhibits the autophagy initiation by preventing the formation of pre-autophagosomal structures (72). The helicase Nsp13 mediates the autophagic degradation of TBK1, impairing IFN-I production and reducing innate immunity (53), while Nsp15, as ORF3a alters early phases of autophagy with the reduction of autophagosome formation (42).

The interplay beween SARS-CoV-2 proteins and the process of autophagy are extensively reviewed in three recent reports (73–75).

Overall, the principal effect of SARS-CoV-2 evasion strategies is the increased viremia which favors the persistent viral stimulation with two main consequences: an increased programmed cell death (PCD) and an increased dysfunction/unbalance of innate immunity.

### 3.1 Increased programmed cell death

Some PCD pathways are upstream of inflammatory processes, playing a critical role in favoring severe outcomes. Cytokines, PAMPs, and DAMPs promote some lytic forms of inflammatory cell death, contributing to fatal evolution of COVID-19 (76–79). For instance, the combination of IFN-γ and TNF-α induces PANoptosis (77), an inflammatory lytic cell death pathway of innate immunity driven by caspases and receptor-interacting protein kinases (RIPKs) that are regulated by multiprotein PANoptosome complexes. The IFN signaling molecules STAT1, IRF-1, NOS2 also promote activation of caspase-8-dependent complex inducing PANoptosis (77, 79, 80).

Proteins induced by IFN-α-activated IFN Signature Genes (ISG) (as ZBP1, AIM2, and ISG-15) may sense viral components forming similar multiprotein complexes leading to PANoptosis (76). In addition, AIM2 recognizing mitochondrial DNA, cell-free DNA, or endogenous DNA, forms another multiprotein complex (AIM2-PANoptosome) leading to PANoptosis. NLR pirin domain containing 1 and 3 (NLRP1/3) and AIM2 bind cytosolic DAMPs and PAMPs and activate the inflammasome, leading to pyroptosis.

PANoptosis induces the death of cells which, in turn, release DAMPs and alarmins engaging PRRs resulting in amplification of inflammation (77, 81). PCD is induced during the entire SARS-CoV-2 progression: initially, the virus infects the upper-airways, sensitizing epithelial cells to cell death (82, 83). Subsequently, it may spreads to alveoli infecting type II pneumocytes and trigger innate cells (84, 85) that undergo pyroptosis (61), releasing PAMPs/DAMPs and cytokines further recruiting and activating other cells (86, 87). Neutrophils mainly undergo neutrophil-extracellular traps (NETs) PCD (82, 88–91). Similarly, PANoptosis contributes to endothelial cell death and organ damage in adults with severe COVID-19 and children with Multisystem inflammatory syndrome (MIS-C) (92), possibly resulting in abnormal blood clots, lung damage, myocardial infarction, and stroke (93).

### 3.2 Dysfunction of innate cells

The virus and soluble S1 can interact with some active receptors/molecules (membrane binding lectins – MBL-, CD26, CD147, CD209, Histamine receptor H1, TLRs) expressed on many cell types by directly or indirectly interfering with functions of the majority of innate- and, subsequently, adaptive cells (94, 95). It also displays sequences with super antigenic- and/or self-antigen-like activity inducing activation of polyclonal- or autoreactive T cells.

Herein we will briefly examine how Virus/S1 protein-receptors interplay can modify the features of infected- and not infected cells of innate immunity during SARS-CoV-2 infection.

Thus, the number of circulating monocytes is reduced, showing an activated immature phenotype that does not result in production of excess of cytokines (96). In contrast, PB DCs appear much more dysfunctional expressing reduced anti-viral ISGs, MHC-Class II antigens and cytolytic activity (97, 98). Lung alveolar macrophages are replaced by inflammatory CD163+monocyte-derived (not tissue resident) macrophages sharing some regulatory activities with myeloid-derived suppressor cells (MDSCs), overexpressing inflammasome-, pro-fibrotic- and C’-related genes and producing pro-inflammatory chemokines and cytokines (99, 100). These molecules start and amplify a vicious loop further promoting lung homing of blood activated monocytes and T (Th17 and cytolytic CD8+) cells, that improve DAMP release and tissue damage, further activation of macrophages with increase of cytokine release (97).

It is not clear if neutrophils can be directly infected (89, 91, 101): TLR engagement activates downstream NF-kB and interferon regulatory factor 7 (IRF7) with the production of proteases, cationic polypeptides and pro-inflammatory cytokines/chemokines. Virus engagement of neutrophil TLR7/8, activates protein arginase deiminase 4 (PAD4), inducing chromatin de-condensation and NET formation (102, 103). NETs trigger a positive loop with macrophages that are activated, produce IL-1β, CCL1, CCL2, IL-6 and TNF-α, and further recruit neutrophils. Extracellular histones from NETs cause cell cytotoxicity promoting ARDS, sepsis and organ failure, while extracellular DNA, favors autoimmunity and, through mucus hyperproduction, bacteria superinfection with respiratory failure. Lastly, NETs release fibrinogen, Von Willebrand Factor (VWF) causing thrombosis in lung, kidney, liver and peripheral vessels (104). Endothelium damage and thrombosis can also be improved by SARS-CoV-2-mediated C’ activation through all three C’ pathways: they are started up S1 and N proteins, producing anaphylatoxins C3a and C5a, whose receptors are present on endothelial cells, platelets and most leukocytes, inducing a prothrombotic state frequently triggering thrombo-inflammatory events (105–107).

Increased mononuclear (M-) (108) and polymorphonuclear (PMN) MDSCs (109, 110) have been reported in COVID-19 patients (111). The MDSC gene signature is predominant in PB from severe patients: M-MDSC number is higher in severe vs mild patients (112), is related to viral load (113) and associated with secondary infections and mortality (114). The reduced/delayed IFN production associates with enhanced chemokines that recruit MDSCs into the lung, while high IL-6 may favor MDSC proliferation (115). PMN-MDSCs use reactive oxygen species (ROS) and L-arginase, whereas M-MDSCs use inducible nitric oxide synthase (iNOS) and L-arginase to suppress bystander immune cells (116). Regulatory functions of MDSCs include i. Induction of high PD-L1 expression decreasing antigen-specific T-cells through interaction with PD-1+ T-cells, ii. increased signaling through Galectin-9 and Tim-3 pathways inhibiting Th1 and CD8+T cells, iii. enhanced TGF-β and IL-10 enhancing suppressive function of M2-macrophages and proliferation of Treg cells, iv. upregulation of TGF-β, ROS and L-arginase inhibiting NK and CD8+ T cells, v. elevated pro-inflammatory cytokines contributing to cytokine storm (117).

Few reports on helper Innate Lymphoid Cells (ILCs) indicated decreased ILC- and ILC precursor subsets in all COVID-19 patients: however, the percentage of ILC2 upon total ILCs is increased in patients vs not infected controls (118, 119). CD117low ILC2, a subset secreting more type 2 cytokines, is expanded in COVID-19 patients as also confirmed by single cell RNA sequencing (scRNAseq) (118, 119). ILC2 and ILC precursors display enhanced CD69 and NKG2D and reduced CXCR3 and CCR4, CD25 and KLRG1 expression (118, 119). At present it is not clear whether ILC changes are related to worse or improved outcomes or may be considered a simple epiphenomenon (101).

The NK cell dysfunction in early infected- or severe patients has been repeatedly reported (118–120), the absolute number being reduced during infection (121) and restored after recovery (122). Patients with severe disease usually show at the early onset increased pro-inflammatory cytokines, including IFN−γ produced by NK cells (123). These cells are strongly activated (106) and highly express inhibitory checkpoint- (LAG3, PD-1, TIM-3) or inhibiting receptors mainly in CD56^dim^ subset, suggesting a dysfunctional/exhausted profile (124), favoring the pathogenesis rather than limiting infection (125). Even though some reports indicated that NK alterations are due to enviromental signals (94, 123, 126), we recently demonstrated that the virus can directly activate NK cells till their exhaustion (127). HLA-E-binding S1 peptide(s) expressed by infected epithelial cells may favor lung homing and recognition of inhibitory CD94/NKG2A+ NK cells (128). NK cell dysfunction also associates with low NK-stimulating cytokines (as IL-12, IL-15) from APCs which, instead, produce IL-10 and TGF-β (129), as virus-stimulated fibroblasts, epithelial and endothelial cells (109). The IL-6 overproduction inhibits *in vitro* NK cell cytotoxicity (130) indirectly confirmed by the *in vivo* treatment with anti-IL-6R mAb which increases NK cell function in COVID-19 patients (131).

Dysfunctional innate cells have a great impact also on the upgrowth of altered adaptive immunity (110). Modified function of APCs impairs the induction of virus-specific T cells which also display lower cytotoxicity and IFN-γ production. M2-type macrophages and MDSCs lead to expanded non cytolytic type 2 cells in tissues (Th2, ILC2, Tfh2, etc) that, at lymph nodal level, stimulate antibody- and, sometimes, autoantibody production by B cells. Increased viral components (expressing superantigens or autoantigens) favor the expansion of non-specific polyclonal- or autoreactive T cells. Lastly, the cytokine milieu (TGF-β, IL-1β, IL-6, IL-23) of tissue inflamed cells favors the development and expansion of Th17 and Treg cells which can switch each other along the infection (126).

## 4 TLR4-SARS-CoV-2 interaction: role for inflammation maintenance

The maintenance of inflammation evolving to critical outcomes is essentially due to vicious circles involving the virus, dysfunctional innate cells and soluble molecules released from cells of the inflamed tissues. SARS-CoV-2 and its soluble proteins activate innate sensors, as TLRs, mostly expressed in innate cells and detecting not only pathogens but also DAMPs (132). TLR engagement initiates downstream signaling cascade, leading to the release of effector molecules such as inflammatory cytokines/chemokines (132). In the chapter below, we will discuss how, among extracellular TLRs, TLR4 plays a crucial role in maintaining SARS-CoV-2 infection.

### 4.1 The virus preferentially activates TLR4 signaling

Several viruses such as the respiratory syncytial-, vesicular stomatitis- and Ebola virus, can directly engage and activate TLR4 through their surface glycoproteins (133). Although the precise mechanism(s) by which these viruses activate TLR4 remains partially unknown, some authors emphasize the role of glycosylation or hydrophobic (hydrophobic pocket of MD-2 linked to TLR4) interactions. In silico studies have demonstrated that soluble S1 protein can bind TLR2 and TLR4, with a higher affinity for TLR4 (134, 135). This prediction was further validated by *in vitro*, *ex vivo*, and *in vivo* experiments clearly indicating that TLR4 is a high-affinity (~300 nM) cognate receptor for the trimeric S glycoprotein, suggesting its role as a mediator of the proinflammatory response in COVID-19 (127, 136–138).

#### 4.1.1 Direct or endotoxin-mediated S1-TLR4 interactions

The S1-TLR4 direct interaction was debated for long time since a computational modeling analysis revealed a high affinity also between LPS and S1 (139), suggesting that endotoxin contamination in recombinant S1 preparations (produced in *E. coli* or human cells) might be responsible for TLR4 engagement and human macrophage activation. According to these authors, the compound Spike/LPS should act synergistically to induce cell activation, while individual components do not (140). Indeed, some evidence suggests that LPS may be involved in the hyperinflammation of SARS-CoV-2 infection: hospitalized severe COVID-19 patients exhibit elevated levels of LPS in circulation, which increase as the disease progresses (141). Moreover, subclinical infections with Gram-negative bacteria or low levels of LPS from the gut could contribute to interactions between LPS and the S protein in infected patients (142).

In odds with these findings, however, many reports underlined that endotoxin contamination is unlikely to be the sole driver for proinflammatory responses reiterating the direct activation of TLR4 by S1 (136). In agreement, our results indicate that a large spectrum of doses of exogeneous LPS did not potentiate the NK cell functions induced by ultrapure S1, but, rather, resulted in a decrease (127). In addition, S1 induces the NK cell release of TNF-α and IFN-γ that enhance the transcription of CD40 which, interacting with its ligand, stabilizes the membrane expression of TLR4 that serves as a receptor of S1 (143). Omicron S protein which exerts stronger binding affinity for TLR4 than the other VOCs, has been reported to bind LPS with reduced affinity compared to other variants (41, 144). Finally, taking into account that LPS directly binds CD14 and MD2, but not TLR4 (145), the recent in silico definition of the fine hydrophobic bonds between residues of S1 and TLR4 and their ability to induce TLR4 dimerization, strongly suggests that, at least in part, S protein binds TLR4 and triggers subsequent signaling (146). Further insights are, however, mandatory to define the exact role of LPS in S1-TLR4 interaction mainly in severe COVID19 patients.

#### 4.1.2 The TLR4 structure and signaling

The structure of TLR4 includes an extracellular leucine rich repeat (LRR) domain, a transmembrane domain, and an intracellular Toll/Interleukin-1 receptor like (TIR) domain interacting with adaptor proteins TIR domain-containing adaptor protein (TIRAP) and TRIF-related adaptor molecule (TRAM) (147). The TLR4 signaling complex consists of cluster of differentiation 14 (CD14), myeloid differentiation factor-2 (MD-2), TLR4, and TIRAP or TRAM that initiate downstream signaling pathways in a dynamic manner. S1 protein from SARS-CoV-2 triggers two pathways, starting with TLR4 transformation and binding with Myeloid differentiation primary response 88 (MyD88) and TIR domain-containing adaptor protein inducing interferon beta (TRIF) proteins. The first pathway (MyD88-dependent) leads to inflammation through the activation of the IRAK4-IRAK1/IRAK2 complex and of TAK1, allowing the degradation of IκBα and favoring the entry of NF-κB into the nucleus to start the transcription of proinflammatory cytokine genes. TAK1 also triggers MAPK pathways with AP1 activation, which is crucial for cell survival and proliferation. The second pathway (via TRIF, MyD88-independent pathway), essential for the antiviral response, induces the activation of TRAF3 and TRAF6 and later of TBK1 of IKKϵ, two enzymes phosphorylating IRF3, which enters the nucleus and begins the transcription of type I IFN genes (ISG) (132).

#### 4.1.3 TLR4 activation by soluble Spike protein

The entire virions, soluble S1 proteins and S1-bound exosomes are involved in TLR4 activation. The presence of soluble S1 is a relatively frequent event in SARS-CoV-2 inflamed environment due to its release from virus-infected and apoptotic cells. The cleavage of SP from each viral particle can produce about 50–100 molecules/virion of soluble S1, that can elicit multiple biological activities (148). High serum S1 levels have been reported during early onset and severe outcomes (124, 125). In post-acute sequelae of SARS-CoV-2 infection (PASC or Long-COVID19) high levels of soluble S1 and TLR4 expression have been described: importantly, in mice and humans, soluble SP may induce TLR4-mediated long-term cognitive dysfunction (135, 149–151). However, there is no evidence that TLR4 activation in PASC persists independently of detectable viral antigens. Clinical studies indicate that antagonizing TLR4 signaling dampens the cytokine storm of severe COVID-19, reduces mortality rates (152, 153), and has therapeutic effects in PASC patients (154).

High serum S1 levels have been also reported in post-vaccination side effects (155): indeed, the S1-coding mRNA, established by replacement of uridine with N1-methylpseudouridine (156) and packaged into lipid nanoparticles, is able to accumulate at the injection site and transported to lymph nodes through DCs. The unprocessed residual vaccine particles are spilled into bloodstream and high S1 levels may persist in blood and tissues for a long time after vaccination. The persistence of soluble S1 in some vaccinated individuals may induce pathogenic processes, being associated with increased expression of TLR4 in specific target cells (157–161).

SARS-CoV-2 can also indirectly activate TLR4 and hyperinflammatory pathways through high plasma levels of DAMPs or alarmins mostly produced by increased apoptosis of infected cells and causing cytokine storm in severe COVID-19 patients (162–165). DAMPs include i. Heat Shock Protein 70 which triggers inflammatory responses during chronic stress through TLR4 (166); ii. S100A8/A9, calcium-binding proteins that activate TLR4–MyD88 pathway (167, 168), thus favoring the output of cells as MDSCs (168); iii. Fibrinogen which stimulates the production of pro-inflammatory cytokines/chemokines in macrophages via TLR4 (169); iv. Secreted Protein, Acidic and Rich in Cysteines-like 1 (SPARCL1) that, through TLR4, induces lung inflammation inducing M1-macrophages and activating the NF-κB pathway (170); v. High Mobility Group Box 1 (HMGB1), released upon necrotic or hypoxic conditions, which promotes inflammation through TLR4 (171). HMGB1 serum level is higher in COVID-19 patients admitted to ICU compared to mild infection (172), whereas SPARCL1 plasma levels are increased in fatal COVID-19 compared to survivors (170).

### 4.2 SARS-CoV-2 infection contributes to upregulation of TLR4

Since peripheral blood mononuclear cells (PBMCs) from COVID-19 patients show enhanced TLR4 expression and phosphorylated NF-κB in circulating monocytes compared to healthy donors (HD) (173, 174), it has been hypothesized that some viral proteins or inflammatory signals may upregulate TLR4 during infection, thus facilitating the amplification of inflammatory circuits. Even though there is no evidence that soluble S1 or S1-bound exosomes can directly upregulate TLR4 after triggering innate cells, other mechanisms indirectly due to SARS-CoV-2 infection have been shown to enhance TLR4 expression.

The first signal is constituted by the decrease of surfactant proteins: the alveolar type II (ATII) pneumocytes represent the targets for SARS-CoV-2 infection due to high co-expression of ACE2 and TMPRSS2 (175, 176). ATII cells are also the sole producers of surfactants, a group of molecules selectively downregulating TLR4 expression on many cell types (177). Surfactant is a mixture of lipids (90%) and proteins (10%), exclusively produced by ATII. Palmitoyl-oleolyl-phosphatidylglycerols (POPG) present in extracellular compartment of alveoli are the molecules most implicated in blocking TLR4 in the lung to avoid untowards proinflammatory effects. The viral induced-ATII apoptosis leads to the decrease of surfactant and the contemporary break of suppressed TLR4 expression (22, 178, 179). Indeed, several reports indicated that reduced levels of POPG in the lung might contribute to develop, or cause, lung diseases as ARDS, because of insufficient suppression of inflammation due to high TLR4 expression and activation (180, 181). The increased TLR4 expression upregulates its interaction with S1, further increasing proinflammatory signaling pathways with enhancement of ISG and ACE2 expression (177). This favors viral replication and innate cell infiltration with a bias towards the signaling pathway leading to hyperinflammation within the alveoli (21). Engagement of overexpressed TLR4 can be considered a critical factor for the severity and mortality of COVID-19 patients, mainly those with comorbidities (177, 182), and for long-COVID-19 cardiac abnormalities (183).

Another signal upregulating TLR4 expression is related to platelet activation (184). Platelets expressing ACE2 and TMPRSS2 are activated by S1 proteins, favoring the release of Tissue Factor converting prothrombin to thrombin and starting clot formation (185). Some reports showed an increased expression of TLR4 on the surface of thrombin-activated platelets of severe COVID-19 patients. Indeed, thrombin can signal through the protease-activated (PAR1 or PAR4) receptors expressed on platelets to induce a phospholipase C (PLC)-dependent intracellular calcium mobilization, which activates calpain and favors intracellular α-granules-containing TLR4 trafficking towards the surface of platelets (186). This event may have relevant inflammatory consequences, since S1 and TLR4 co-localize on platelets isolated from COVID-19 patients with aggregated platelets and thrombus growth (186). Additionally, SARS-CoV-2-containing S100A8/A9+megakaryocytes, exhibit high TLR4 surface expression that correlates with NF-κB activation and the levels of released IL-6 and IL-1β (187). These cells are considered significant risk factors for mortality and multiorgan injury in COVID-19 patients (187). The platelet activation by S1 and the subsequent TLR4 overexpression are responsible for the thrombophilia state associated with severe outcomes (184).

A third mechanism upregulating TLR4 expression is the direct consequence of S1-ACE2 interaction which is usually associated with downregulation of ACE2 via different mechanisms (188). ACE2 reduction favors the increase of angiotensin II (AngII) which, by interacting with AngII type 1 receptors (AT1-R), induces vasoconstriction, hypoxemia, increased endothelial injury, and tissue necrosis which further contribute to increase both inflammation and diffuse thromboembolic effects (189, 190). Downstream signaling as receptor tyrosine kinases, NF-κB, MAP Kinases and, above all, the upregulation of TLR4 are linked to the AngII-AT1-R interaction, especially in SARS-CoV-2-related cardiovascular diseases (191). Interestingly, the increase of TLR4/NF-κB signaling and cytokines by M1 macrophages following AngII-AT1-R interaction have been largely confirmed in animal models (192).

Finally, the elevated testosterone levels and some TLR4 polymorphisms could also be implicated in the upregulation of TLR4.

### 4.3 TLR4 activation of immune- and non Immune cells

#### 4.3.1 Immune cells

The viral infection has a deep impact on the increase of TLR4 expression on innate (macrophages, DC, neutrophils, NK) immune and non-immune (neurons, epithelial) cells, which, in turn, upgrow inflammatory responses through the same receptor (137).

#### 4.3.2 Macrophages/DCs

S1-TLR4 interplay can primarily activate macrophages/DCs in murine models leading to the release of inflammatory mediators such as IL-1β, TNF-α, IL-6 and nitric oxide through the NF-kB and JNK signaling pathways, which are deeply suppressed with specific antagonists. In agreement, the proinflammatory response to S1 was abrogated in macrophages from TLR4−/− mice (136) or with siRNA targeting TLR4 (193). TLR4 engagement favors ACE2 expression, viral entry and hyperinflammation of macrophages, playing the major role in amplifying inflammatory circuits during severe COVID-19 (194).

#### 4.3.3 Neutrophils

TLR4 also contributes to the formation of neutrophil extracellular traps (NETs) that exacerbates and prolongs the deleterious proinflammatory environment in severe patients (195). TLR4-induced expression of ACE2 was higher in the myeloid cells of severe COVID-19 patients and was associated with elevated levels of the immune checkpoint molecule PD-L1 that can suppress antiviral T cell response upon interaction with PD-1 on effector cells (196, 197).

#### 4.3.4 NK cells

**Figure 1:**
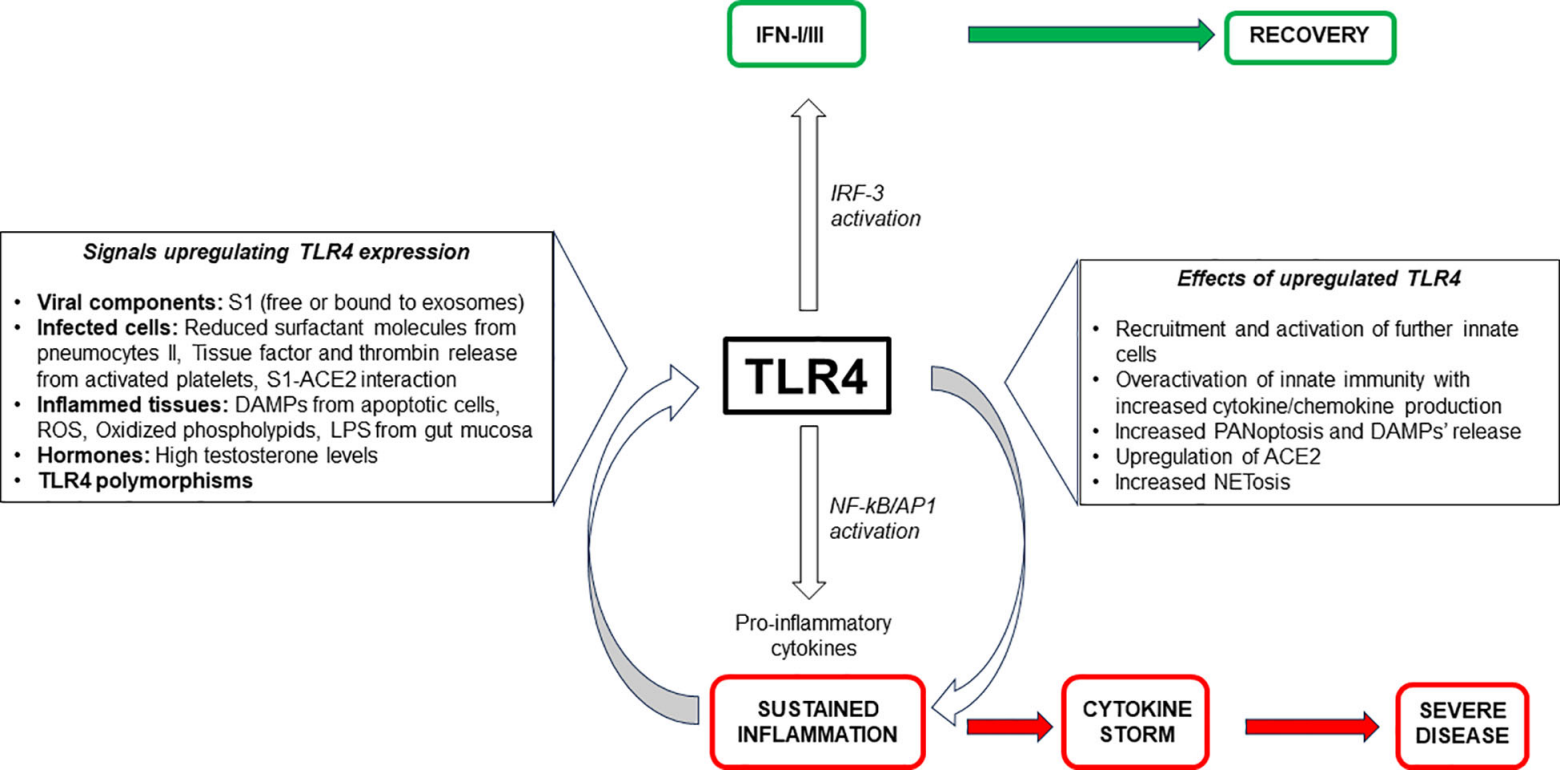
**(A)** Protective Immunity loops to fight SARS-CoV-2 infection. **(B)** Persistent viral load and high soluble S1 protein impair innate immunity leading to detrimental effects.

Furthermore, S1-TLR4 interaction involves directly also NK cells. We have recently shown that S1 from the Wuhan strain and other VOCs bind and activate TLR4 (and less TLR2) on purified PB NK cells by increasing phosphorylation of NFkB, activation marker expression, cytokine release, and cytolytic activity (127). Of note, S1-TLR2/4 interaction does not trigger ACE2 on NK cells or their activating receptors (DNAM1, NKGD2 Nkp30/44, etc) (127). Recently recovered patients displayed a higher proportion of circulating NK cells (vs HD) which can be stimulated *in vitro* by S1. This likely explains why NK cells are currently highly activated *in vivo* during infection and recovery. In addition, S1 significantly amplified *in vitro* NKG2C+CD56^dim^ NK cells, a phenotype typical of “trained cells” (127). In agreement, some studies observed that increased adaptive response is followed by expansion of exhausted NKG2C^+^CD57^+^NK cells (198, 199). Notably, since signals inducing trained immunity, such as BCG, initiate their activity via TLR2/TLR4 engagement, it is conceivable that S1 induces expansion of trained NK cells through a similar mechanism (200). It is likely that S1-driven NK cell activation can induce initially the amplification of protective trained (NKG2C+CD56^dim^) cells favoring cytolytic activity of infected cells and upregulation of IFN-γ activating a response characterized by M1- and Th1 cells. Persistent viral load and chronic S1 stimulation may, however, lead to exhaustion of NK cells downregulating cytotoxicity and IFN-γ production and favoring sustained inflammation and viral spread (**Figures 1A, B**).

#### 4.3.5 Non-immune cells

Beyond the effects on endothelial cells, TLR4 triggering has also been shown to directly mediate neuroinflammation or renal tubular epithelial cell damages.

Indeed, in murine models S1 induces neuro-inflammation and memory dysfunction in post-COVID-19 syndrome through TLR4 pathway (149, 201). Other *in vivo* studies have revealed the possibility that S1 may induce neuroinflammation and memory dysfunction through TLR4-expressing microglial cells and neurons (149). The pediatric cerebral cortical neuronal cell line (HCN-2) lacking ACE2 exhibited elevated TLR4 transcript levels alongside increased secretion of proinflammatory cytokines and chemokines, indicating that TLR4 can mediate neurological effects (202). Remarkably, by crossing the blood–brain barrier S1 triggers neuroinflammation thus supporting the hypothesis that the virus may produce comparable effects in human Long COVID-19 patients experiencing cognitive dysfunction (203).

Lastly, it has been shown that *TLR4* is one of the risk genes associated with immune-inflammation-promoting renal injury in severe COVID-19 patients (204). By using human kidney cell lines, it has been demonstrated that SARS-CoV-2 directly induces damage of renal tubular epithelial cells via TLR4 and IL-1R signaling (205). Moreover, TLR4 contributes to kidney damage favoring SARS-CoV-2-induced inhibition of albumin endocytosis through decreased Akt activity in proximal tubule epithelial cells (206).

### 4.4 Population genetics and comorbilities

TLR4 polymorphisms can also play a role in the pathogenesis of COVID-19. Human TLR4 presents two notable single nucleotide polymorphisms (SNPs)—896 A/G and 1196 C/T—favoring COVID-19 severity (207). On the other hand, the 896 A/G variant has recently been identified as a protective factor against COVID-19 progression among younger individuals without cardiovascular abnormalities (208). Patients carrying the 1196 C/T SNP develop more frequently pneumonia, leading to critical manifestations (209), whereas the rs4986790 (896) GG genotype displays a defective TLR4 signaling leading to cellular dysfunction, associated with severe disease (210). Other TLR4 polymorphisms linked to COVID-19 severity as SNP -2604G>A has been associated with increased neuroinflammation and cognitive dysfunction (149). TLR4 polymorphism is likely to be involved also in Long-COVID-19: 52% of severe long-COVID patients carried at least one disease signature variant in TLR4 (154). Lastly, beyond genetic polymorphisms, TLR4 expression is higher in men vs women due to testosterone levels, correlating with elevated pro-inflammatory cytokine levels, mainly in COVID-19 (195).

**Figure 2:**
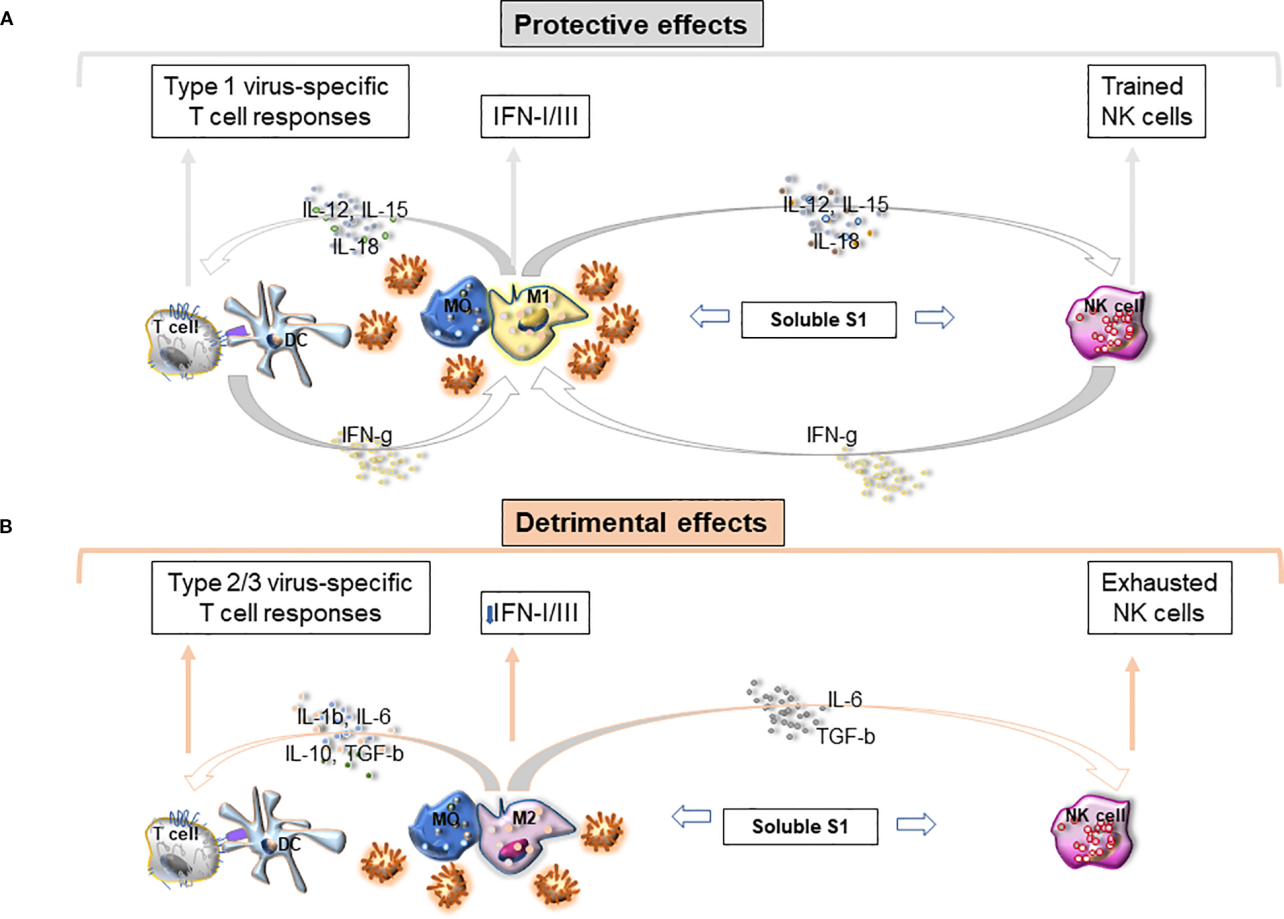
Role of TLR4 expression and signaling on innate cells in the pathogenesis of severe COVID-19.

Some recent reports on the quantitative analysis of public transcriptomic datasets on TLR4 expression levels in COVID 19 have been published, even though they do not relate it to the disease severity. By comparing two or three gene expression datasets and performing bioinformatic methods to construct protein-protein interaction (PPI) networks, in three separate reports TLR4 always resulted among the hub genes more expressed (211–213). By contrast, metanalyses on TLR4 expression in COVID-19 are at present lacking. The role of TLR4 hyperexpression and signaling on innate immune cells is summarized on **Figure 2**.

### 4.5 Clinical relevance of S1-TLR4 interplay

Clearcut results indicated that TLR4 is broadly upregulated in COVID-19 patients and participates in various COVID-19-related pathologies. TLR4 (and less TLR2) expression is upregulated in PBMCs or BALF from severe patients compared to mild cases or HD (173, 214). Critical COVID-19 patients exhibit higher levels of TLR4 and phosphorylated NF-κB in CD14+ HLA-DR^high^ circulating monocytes, with increased NF-κB p65 phosphorylation in the CD14+ HLA-DR^low^ monocyte subset (174, 215). Moreover, severe patients exhibited a two-fold increase of TLR4 expression in nasopharyngeal cell samples as compared to patients with mild disease (216) and TLR4 plasma levels correlated positively with COVID-19 severity (217). Lastly, in the autopsy of COVID-19 patients a massive TLR4 upregulation in the lung was associated with increased macrophage infiltration, presenting a shift from GAL-3+ alveolar macrophages to CD163+ myeloid-derived monocyte-macrophages: even though these results do not show any direct cause/effect relationship, however, they indicate that TLR4 expression may induce a persistent inflammation, with inefficient resolution, and pathological macrophage shift which could be one of the mechanisms of lethal COVID-19 (218). Notably, COVID-19 non-survivors have higher plasma levels of LPS (the most usual TLR4 ligand) than survivors, due to virus-related intestinal permeabilization and translocation into the blood of enteric pathogens or their products (141). Hospitalized severe COVID-19 patients also display elevated LPS levels increasing with the disease progression, thus confirming that the endotoxin itself may play a role in SARS-CoV-2 hyperinflammation (141). Finally, TLR4 activation also exhibited reduced cytokine secretion from monocytes of convalescent COVID-19 patients (219). These data suggest that following SARS-CoV-2 infection, chronically stimulated monocytes exhibit exhausted steady-state gene expression and reduced responsiveness. This may be also due to the increased levels of soluble TLR4 and CD14 acting as decoy receptors (probably released upon an excess of TLR4 activation) which decrease TLR4-mediated signaling and inflammatory responses (220). A clinical consequence of a sustained decrease in the response of these PRRs could also be an increased susceptibility to other unrelated infections or superinfection with other pathogens (221, 222).

Lastly, as previously underlined, TLR4 is also involved in SARS-CoV-2-associated extra-pulmonary immune-pathologies as the kidney injury and the neurological symptoms (149, 206).

### 4.6 Central role of TLR4 to orient the outcomes of infection

TLR4 does not fully encompass the disease’s complex and intricate immune mechanisms of SARS-CoV-2 infection, involving a wide network of immune sensors and pathways. However, the TLR4 pathway plays a significant role in severe inflammation since, as described, the inflammatory signals enhancing TLR4 expression make TLR4-bearing cells more susceptible to triggering by viral components, thus increasing and maintaining inflammation (22). Thus, it is possible to speculate on a dual role of TLR4, both protective and deleterious, depending on the phase of the disease. As long as the type I IFN induced by the TLR4 signaling pathway remains unmodified, the antiviral response is effective; the viral load is at low levels and clinical remission occurs. In this phase S1-activated TLR4+ NK cells favor the antiviral effects by increasing their function, mounting a “trained immunity” response and contributing to protection towards the virus. However, when the evasion strategies of the virus mainly impairing IFN release and activity, predominates, the viral load increases and the pro-inflammatory responses induced by TLR4 signaling greatly prevail. Under these conditions a vicious circle is established essentially due to multiple mechanisms increasing TLR4 expression and its active signaling. In the absence of a valid IFN response, the upregulation of TLR4, in turn, stimulates ACE2 expression, NETosis and PANoptosis, with a substantial increase of cytokines/chemokines recruiting new circulating cells. In this phase persistent S1 activation of NK cells through TLR4 leads to cell exhaustion and consequentially to the enhancement of viral load. Such events facilitate stimulation of macrophages by viral S1 and DAMPs which further upregulate TLR4, thus creating a feedback loop, where heightened TLR4 levels increase accessibility to S protein leading to the inflammation maintenance and favoring severe outcomes. According with some authors TLR4 can be considered a critical “fate-deciding molecule” for the pathogenesis of severe COVID-19 (22, 153, 179) (**Figure 2**).

## 5 Targeting TLR4 is a novel therapeutic option for SARS-CoV-2 infection

By considering the TLR4 role in immune response and disease pathogenesis, molecules or vaccines targeting TLR4 may provide a therapeuticl option for SARS-CoV-2 and for the majority of huCoV infections (22). A complete and exhaustive review on TLR4 agonists and antagonists and drugs interfering with TLR4-S1 interaction has been recently reported (21). It is important to underline, however, that few papers are at present available on TLR4 inhibition in animal models of severe SARS-CoV-1 infection and COVID-19 (223, 224). In addition, the majority of these approaches often failed when employed in other types of diseases such as sepsis and ischemic stroke (225–227).

A variety of natural products, particularly biomacromolecules (LPS from the bacterium *Rhodobacter* sp*haeroides* and TLR4-binding peptide derived from Bacillus-fermented soybean), have been investigated as alternative options to block TLR4 or disrupt downstream signaling pathways (228). Phytochemicals, such as jacareubin, cajastelebenic acid, andrographolide, cannabidiol, and berberine, have been documented as potent blockers of TLR4, some also being validated with clinical trials (229). A series of chemically synthesized compounds and peptides have been identified for their ability to interfere with TLR4 activity that might help in combating COVID-19. For example, TLR4 antagonists such as Eritoran sulfate (E5564) and FP7 have shown efficacy in reducing lethal damage associated with severe influenza and sepsis (230).

Other potentially useful compounds are Opioids (naloxone, naltrexone, and tramadol), which exhibit TLR4-antagonizing properties. Many small molecules (as Disulfiram, dimethyl fumarate, fluoroquinolone antibiotics) that directly or indirectly antagonize TLR4 are also in development or undergoing preclinical validation. Some TLR4 agonists and antagonists have reached various phases of clinical trials, including peptides (EC-18), chemical compounds like imiquimod, hydroxychloroquine, and artesunate, DPP4 inhibitors or small molecules (as PUL-042) (153).

Moreover, the administration of probiotic bacterial strains has emerged as a promising approach to impair the harmful effects of TLR4 activation with potential benefit in COVID-19 patients. Genetically engineered probiotic (bacterium *Lactobacillus paracasei* F19 producing palmitoyl-ethanolamide) has been intranasal administered resulting effective in reducing SARS-CoV-2-associated lung injury by blocking TLR4- mediated NLRP3 activation and decreasing pro- inflammatory cytokines (231).

Alternative strategies targeting TLR4 include monoclonal antibodies (mAbs) with inhibitory activity (232). For instance, paridiprubart (EB05) prevents TLR4 dimer formation, thereby blocking the response to TLR4 agonists such as S1. Importantly, this compound resulted in 100% survival in coronavirus mouse model (168). It has potential for yielding similar beneficial effects in impairing the extreme inflammatory response observed in Interstitial lung fibrosis and ARDS. Even though approved for advanced phase 3 trials, unfortunately they were suspended or ended inconclusively for the lack of patient recruitment (https://clinicaltrials.gov/study/NCT04401475, https://clinicaltrials.gov/study/NCT05293236). Since many VOCs and their subvariants have developed resistance to mAb treatments, the design of chimeric mAbs incorporating complementarity-determining regions (CDRs) from regdanivimab and sotrovimab, or from bebtelovimab and adintrevimab, has been proposed (233–235).

Aptamers are a further innovative approach to target TLR4 in COVID-19 since they: i. are small size, single-stranded DNA or RNA, molecules folded into unique 3D structures, ii. are specifically bound to target molecules with high affinity (236), usually displaying less steric hindrance and better access to binding sites compared to antibodies, iii. are of easy synthesis, low immunogenicity, and useful in detection and therapy. APToll is a notable aptamer currently in clinical use for cerebral ischemic events, demonstrating the potential benefit of aptamer-based therapies (236).

The emergence of new VOCs often evading the protection provided by the antibody-induced response elicited by Spike-based vaccines imposes to develop new type of vaccines based on pathogenetic, poorly mutagenic molecular structures (153). In the AbhiSCoVac vaccine the constructed peptide is designed to stably engage major immune sensors like TLR4, TLR2, MHC class I and II (237). Due the Spike-TLR4 interaction a common feature of huCVs, studies are presently focused to identify specific sequences responsible for this interaction, to develop a multi-epitope multi-target chimeric vaccine effective against not only SARS-CoV-2 VOCs but also virtually all huCVs (237).

**Table 2** summarizes the most recent therapeutic approaches antagonizing TLR4 engagement and signaling.

**Table 2:** Therapeutical compounds to inhibit TLR4 engagement or its signaling.

Therapy antagonizing TLR4 signaling	Reagent	Function	Pathogenic activity	Clinical trials	References
Vaccines	AbhiSCoVac	Multi-epitopic, multi-targeted chimeric vaccine (Constructed Peptide) to stable engage TLR4, TLR2, MHC Class I/II	To generate protective immunity against all six virulent members of the huCoV family	In vivo and in silico studies	(21, 153, 237)
Peptide/mAbs	Paridiprubart (EB05)	Peptide blocking dimerization of TLRs, blocking S1-TLR4 interaction	To block LPS-induced IL-6 release	NCT04401475 Phase 2 (in progress)	(21)
	Chimeric EB05 or other anti-TLR4 mAb (NI0101) linked to an anti-CDR mAbs	To block Spike-TLR4 interaction		Proposed for new pathogenic VOCs	(21, 225, 233–235)
Bio-Macro molecules	Lung Surfactant-BL		To decrease cytokine storm and ARDS in severe COVID19 patients	NCT04568018 Open-labeled Observational trial	(21)
	LPS from the bacterium *Rhodobacter sphaeroides*, TLR4-binding peptide from Bacillus-fermented soybeans				(21, 227, 228, 250)
Repurposed Drugs/ Synthetic molecules	Linagliptin	DPP4 and cytokine inhibitor	To decrease cytokine storm in severe patients	NCT04341935 Case control study	(251)
	Eritoran (mimicking Lipid A)		To ameliorate community-acquired pneumonia in CAVID19 patients	NCT02735707 Phase 3 trial	(226, 230)
	Opioids (Naltrexone, naloxone)	TLR4 antagonizing activity	To reduce pathologic outcomes of COVID-19	NCT04604704 Phase 2 trial	(21, 252)
	Opioid (Tramadol)		To reduce inflammation and hypercoagulability	NCT04454307 Phase 1/2 DBC trial	(21)
	Small Molecules (Disulfiram, dimethyl fumarate, fluoroquinolone antibiotics)	To inhibit TLR4 (directly or indirectly)			(253, 254)
Aptamers	ApTOLL	High affinity small size single- stranded RNAs or DNAs bound to targets	To block cytokine storm in infected patients	NCT05293236(Stopped since COVID-19 cases drastically reduced in Spain)	(236)
Phytochemicals	Jacareubin, Cajastelebenic acid, Andrographolide, Cannabidiol, Berberine				(21, 229) (229, 255, 256)

In conclusion, even though some data are highly promising, more studies are needed to define how any proposed TLR4-related therapeutic strategies would be relevant in real world practice, due to the complexities (as host-genetic polymorphisms, VOC-specific immune engagement, correct timing and duration of treatment) and risks (potential losing antiviral and even a general immune protection) to interfering with such an innate fundamental receptor.

## 6 Conclusions

The innate immune system is the first line of defense against infections, including SARS-CoV-2. In this review we have examined cells and molecules of the innate immunity playing a critical role in the early phase of infection and conditioning the subsequent adaptive response and clinical outcome towards recovery. In this phase, TLRs has a beneficial effect, since the early and strong protective TLR-mediated innate immune response against viruses or viral components is essential for viral clearance, though the secretion of antiviral cytokines, chemokines and type I IFNs.

However, the virus exploits evasion strategies to counteract the innate response prevalently through the inhibition of type I/III IFN and autophagic mechanisms: this leads to C’ overactivation, hyperinflammation, pan-apoptosis and increased viral load which exert a deep dysfunctional impact on cells of both innate- and adaptive immunity.

The maintenance of SARS-CoV-2-related inflammation evolving into critical outcomes is essentially sustained by vicious loops involving dysfunctional innate cells and signals from inflamed environmental cells. In this phase TLRs may be harmful for SARS-CoV-2 infection eliciting dysregulated immune signaling: the excessive TLR activation due to overstimulation by viral proteins or DAMPs released from apoptotic cells can lead to the untoward production of proinflammatory cytokines and chemokines, resulting in severe disease.

In this context the principal vicious circle involves in particular TLR4, which is selectively engaged by S1 protein. These receptors are triggered not only on all APC but also on NK cells: S1 protein strongly increases the function of these cells, selectively expanding initially NKG2C+NK (trained) cells. The persistent S1 stimulation by soluble protein which may be elevated along the infection and in Long-COVID-19, turns this protective mechanism into a progressive exhaustion which increases inflammation and favors virus persistence. The viral excess (S1 protein and Nsps blocking IFN and protective mechanisms) plus various inflammatory signals upregulating TLR4 on innate cells creates a vicious circle maintaining and further enhancing hyperinflammation mediated primarily by monocytes and macrophages, leading to severe or even fatal outcomes. Thus, TLR-dependent anti-viral response or excessive inflammation may tip the balance towards the former or the latter, altering the equilibrium that drives the severity of disease (216). For these reasons, therapeutics targeting the TLR4 signaling pathway may be a promising strategy, potentially offering the dual benefits of viral suppression and inflammation shutdown. Persistent inflammation and immune dysregulation sustained by TLR4 involvement are thought to play an important role also in the case of Long-Covid-19 (238). Pharmacologic agents targeting TLR4 could help in rebalancing the immune system, reducing the likelihood of autoimmune-like conditions observed in these patients (239). Thus, TLR4 inhibitors not only offer a means to mitigate the acute inflammatory response during the initial infection but also provide an option to address the long-term sequelae of COVID-19, mitigating symptoms and accelerating patient recovery.
